# The Cloacal Microbiome of Five Wild Duck Species Varies by Species and Influenza A Virus Infection Status

**DOI:** 10.1128/mSphere.00382-18

**Published:** 2018-10-24

**Authors:** Sarah M. Hird, Holly Ganz, Jonathan A. Eisen, Walter M. Boyce

**Affiliations:** a Department of Molecular and Cell Biology, University of Connecticut, Storrs, Connecticut, USA; b AnimalBiome, Oakland, California, USA; c Genome Center, University of California, Davis, Davis, California, USA; d Pathology, Microbiology and Immunology, University of California, Davis, Davis, California, USA; The Jackson Laboratory for Genomic Medicine

**Keywords:** avian microbiome, ducks, evolutionary biology, microbial ecology, microbiome

## Abstract

Waterfowl, especially ducks of the genus *Anas*, are natural reservoir species for influenza A virus (IAV). Duck populations contain nearly all the known diversity of IAVs, and the birds are asymptomatic to infection. Previous work established that IAV infection status is correlated with changes in the cloacal microbiome in juvenile mallards. Here, we analyze five *Anas* species to determine whether these duck species have similar IAV^+^ and IAV^−^ cloacal microbiomes, or if the relationships among a host, influenza virus, and the microbiome are species specific. We assessed taxonomic composition of the microbiome, alpha diversity, and beta diversity and found very few patterns related to microbiome and infection status across species, while detecting strong differences within species. A host species-specific signal was stronger in IAV^−^ ducks than IAV^+^ ducks, and the effect size of host species on the microbiome was three times higher in IAV^−^ birds than IAV^+^ birds. The mallards and the northern shovelers, the species with highest sample sizes but also with differing feeding ecology, showed especially contrasting patterns in microbiome composition, alpha diversity, and beta diversity. Our results indicate that the microbiome may have a unique relationship with influenza virus infection at the species level.

**IMPORTANCE** Waterfowl are natural reservoir species for influenza A virus (IAV). Thus, they maintain high levels of pathogen diversity, are asymptomatic to the infection, and also contribute to the risk of a global influenza pandemic. An individual’s microbiome is a critical part in how a vertebrate manages pathogens and illness. Here, we describe the cloacal microbiome of 300 wild ducks, from five species (four with previously undescribed microbiomes), including both IAV-negative and IAV-positive individuals. We demonstrate that there is not one consistent “flu-like” microbiome or response to flu across species. Individual duck species appear to have unique relationships between their microbiomes and IAV, and IAV-negative birds have a stronger tie to host species than the IAV-positive birds. In a broad context, understanding the role of the microbiome in IAV reservoir species may have future implications for avian disease management.

## Abstract (precis)

Waterfowl are natural reservoir species for influenza A virus (IAV). Thus, they maintain high levels of pathogen diversity, are asymptomatic to the infection, and also contribute to the risk of a global influenza pandemic. An individual’s microbiome is a critical part in how a vertebrate manages pathogens and illness. Here, we describe the cloacal microbiome of 300 wild ducks, from five species (four with previously undescribed microbiomes), including both IAV-negative and IAV-positive individuals. We demonstrate that there is not one consistent “flu-like” microbiome or response to flu across species. Individual duck species appear to have unique relationships between their microbiomes and IAV, and IAV-negative birds have a stronger tie to host species than the IAV-positive birds. In a broad context, understanding the role of the microbiome in IAV reservoir species may have future implications for avian disease management.

## INTRODUCTION

A vertebrate’s microbiome consists of millions to trillions of microorganisms that exist symbiotically with the host’s immune system, yet all vertebrates must also recognize and combat microbial pathogens. How the microbiome interacts with pathogens is of increasing interest due to recently established causal relationships. For example, a host’s microbiome provides a barrier to incoming pathogens and disruption of this barrier increases colonization by pathogens (1). Pathogens can influence the microbiome through systemic inflammation (such as in HIV infection, reviewed in reference 2) or the altering of microenvironments in local tissue. Changes in the microbiome to a state of dysbiosis can simultaneously be the result of viral infection and the cause of increased susceptibility to pathogens (3).

One globally important virus is influenza A virus (here IAV, family Orthomyxoviridae), which causes significant annual illness and mortality in humans and animals, especially poultry. The IAV genome consists of eight single-stranded RNA segments that code for up to 17 proteins (4). Two glycoproteins play a key role in the ability of the virus to infect a cell. Hemagglutinin (HA) allows the virus to get into a cell, and neuraminidase (NA) allows progeny virus to get out of the cell. The HA and NA subtypes of a virus designate the HA-NA subtype (e.g., H1N1). Birds, specifically waterfowl and shorebirds, are the natural reservoir species for IAV, as nearly all known HA (H1 to H16) and NA (N1 to N9) diversity can be found in their populations, including most subtypes (5–7). Additionally, infection with low-pathogenic avian influenza, or LPAI, causes little to no pathology in waterfowl (7, 8), although LPAI infection may be associated with lower body mass in mallards (9) and altered host behaviors, including feeding and timing of migration, in Bewick’s swans (10). Highly pathogenic avian influenza (HPAI) viruses can evolve in poultry infected with LPAI of H5 or H7 subtypes (11). HPAI infections have more aggressive pathology and higher mortality rates and infect respiratory tissues and other organs, instead of just the intestinal epithelium, as is the case in LPAI (12). HPAI viruses are highly infectious for poultry and can be passed to wild waterfowl and humans (13–16). HPAI can cause illness and mortality in some wild waterfowl species (12), which is concerning because birds migrate and represent a globally connected network of IAV hosts (6, 17). Species common to major flyways (e.g., American and Eurasian), such as Anas acuta (northern pintail), may provide an “intercontinental bridge” for IAVs (6), and their tendency to cross continents during migration is correlated with the amount of mixture between viruses of Eurasian origin and American origin (18, 19). IAVs can cross species and even class boundaries within the Vertebrata, infecting many birds and mammals, including humans (20, 21).

**FIG 1:**
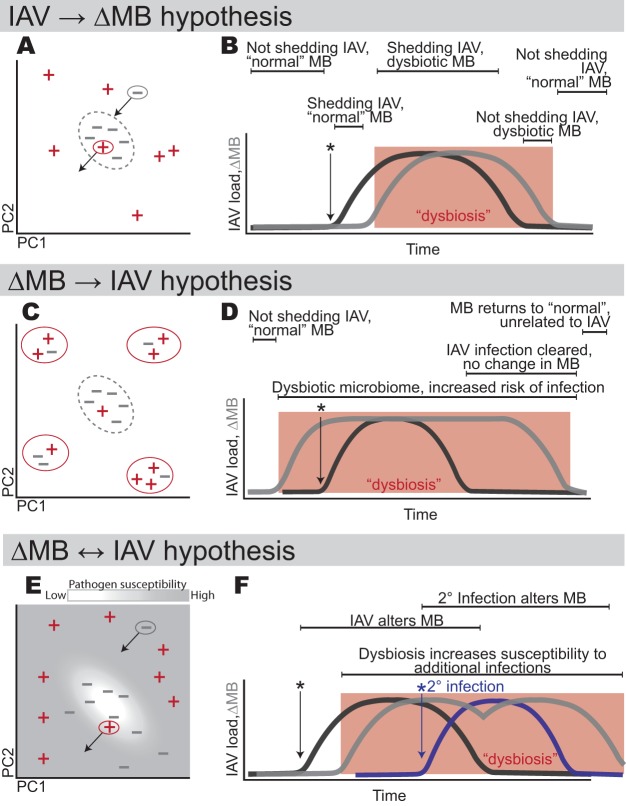
Conceptual model of the relationship between IAV infection and the microbiome. (A) The IAV→ΔMB hypothesis, depicted using a hypothetical ordination showing a central group of “normal” duck microbiomes (−) and IAV^+^ (+) samples surrounding the normal group. The “−” with the arrow shows that as the infection is cleared, the dysbiotic samples will move closer to the normal group as the microbiome recovers. Similarly, the “+” with the arrow shows that a newly infected duck will move further from “normal” as the infection progresses. (B) Over time, the viral load of IAV will increase, and shortly thereafter, the microbiome will also change. As the infection begins to clear, the microbiome will return to the “normal” state. (C) The ΔMB→IAV hypothesis. A dysbiotic microbiome (outlined in red circles) may make ducks more susceptible to infection, but infection itself will not alter the microbiome. (D) As IAV is contracted, proliferates, and is cleared by the host immune system, the microbiome is unaffected. (E) The ΔMB↔IAV hypothesis. Here, the landscape of pathogen susceptibility varies, and species-specific microbiomes have the lowest susceptibility. (F) Dysbiosis makes the host more susceptible to secondary infection, and additional infections further alter the microbiome (i.e., maintain dysbiosis).

Correlative studies, like this one, can be a first step toward establishing causation between dysbiosis and disease. Given a correlation between viral infection and microbiome dysbiosis, one causal hypothesis is that infection by a pathogen can directly cause dysbiosis in the microbiome, the “IAV→ΔMB” hypothesis (Fig. 1A and B). This hypothesis has been demonstrated between IAV and the intestinal microbiota of chickens using time series data from experimentally infected hosts (22). Notably, chickens experience IAV infection more strongly than wild ducks (with some HPAI subtypes having mortality rates of up to 100% in chickens [11, 23, 24]), and the viral infection dynamics are not identical in the two systems. A second hypothesis is that hosts with an altered or dysbiotic microbiome are more prone to pathogen infection (e.g., see reference 25), the “ΔMB→IAV” hypothesis (Fig. 1C and D). Perhaps existing in mixed flocks can alter a wild duck’s microbiome, thus also altering susceptibility to infection. Third, it is possible that both mechanisms are occurring: dysbiotic states can be caused by infection while simultaneously increasing the susceptibility to pathogen invasion, the “ΔMB↔IAV” hypothesis (Fig. 1E and F). This hypothesis has been demonstrated with IAV in a mouse model (3), and overall, it may be the most plausible hypothesis as it acknowledges the various feedbacks and cross talk between a host’s immune system and the microbiome (26).

The relationship between IAV and the microbiome is complex in wild ducks because many extrinsic factors are known to influence both the microbiome and virus dynamics. Many factors contribute to the dynamics of IAV infection in wild birds, including biotic and abiotic properties of a locality, time, space, and the host (27). Genetic similarity of LPAI viruses correlates with geographic space across duck species (28). The virus is likely spread through water, by virions that originated in the feces of an infected duck (29), or through virus shedding from the oropharynx (30). Viruses can persist in water for up to a month, and perhaps longer, depending on the starting viral load, temperature, pH, and salinity of the water (29, 31, 32). Infectious IAV can be isolated from feathers after an even longer time (33). IAV can also persist in sediments and lake ice and may be able to overwinter in aquatic environments (29). Aquatic plants, invertebrates, and filter feeders are also involved in the persistence of IAV in aquatic environments, in some cases sequestering virus in the environment (34, 35).

Additionally, there are influences of space, time, and the host’s immune system on viral system dynamics. Infection in ducks is temporally cyclic, with prevalence of infection, as well as particular subtypes, showing annual patterns that can vary by over 25% between years (36). The cyclical nature of subtypes may be due to the interaction between IAV subtypes and the avian immune system. Infection by a subtype can cause homosubtypic as well as heterosubtypic immunity (37), making reinfection by that subtype in migrating ducks returning to a locality less likely in the following year (although individual immunity varies [38]). The host can also influence virus transmission: age of infection contributes to the amount of virus shed during infection (39), and juveniles who have not been exposed previously typically have the highest prevalence. Host species is also important, as different ducks vary substantially in infection prevalence (6) and viral shedding (40).

Recent work on the microbiome has revealed its important role in health and disease for many vertebrates. How bacteria and viruses interact within the microbiome and how those interactions relate to pathogenicity are active areas of research (41, 42). Many of the same factors that influence flu dynamics are important for wild avian microbiome composition, including age (43), species (44), and sampling locality (45). Migratory status may also influence host health and has been associated with the microbiome as well (46). Understanding how these variables interact across complex, wild systems is important for evolutionary biology, infectious disease studies, and possibly global health.

Previously, we demonstrated that there is a strong correlation between the cloacal microbiome and influenza virus infection status in juvenile mallards from a single collecting locality (47). Ganz et al. (47) were able to detect robust associations between IAV and the microbiome because the study controlled for age, sampling locality, and host species. Here, we investigate the dynamics of IAV infection in five duck species (including the 122 mallards from reference 47) across geographic space and time, with the intention of elucidating the roles of these variables in the cloacal microbiome of IAV^+^ and IAV^−^ ducks. We were particularly interested in whether the cloacal microbiomes of different species bear similar signatures of influenza virus infection status.

Despite the number of potentially confounded variables in our data set, we had several expectations for signal in the data. First, we expected to see decreases in alpha diversity for the IAV^+^ ducks within each species and across the data set, due to influenza virus infection being a state of dysbiosis. Second, we expected there to be compositional differences between the microbiomes of IAV^−^ and IAV^+^ individuals within a species. Changes shared across species were expected to indicate flu-mediated changes. Finally, we expected to detect some taxa (OTUs) that were associated with influenza virus infection, within and across host species.

## RESULTS

### Sequencing.

**FIG 2:**
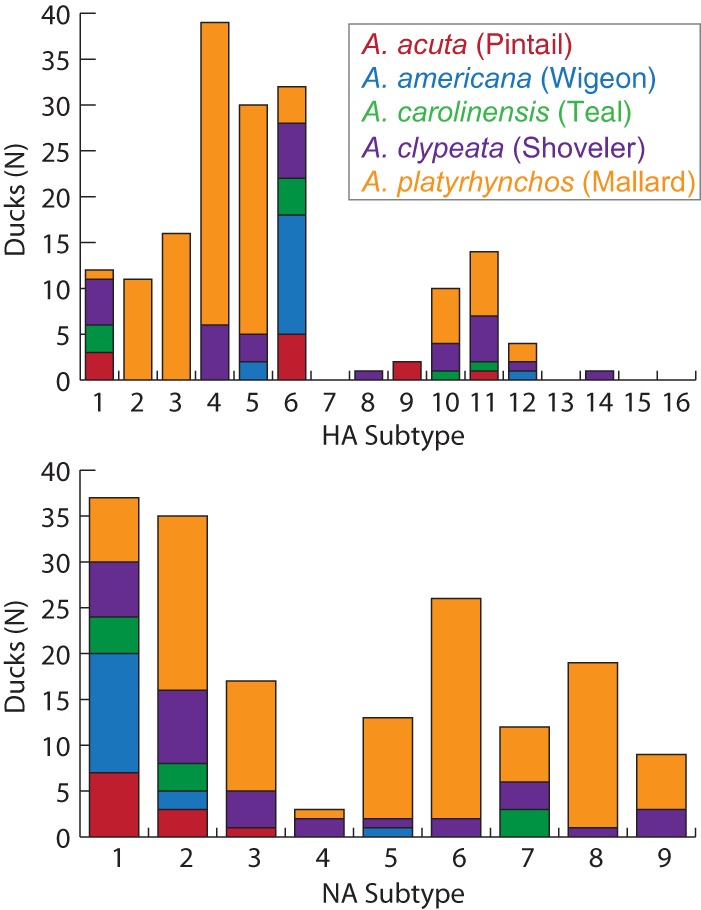
Raw counts of influenza A virus subtypes from the different duck species. (Top) Hemagglutinin (HA) subtype frequencies. (Bottom) Neuraminidase (NA) subtype frequencies.

A total of 10,158,296 reads were produced from 300 birds, belonging to five species (Table 1 and also Table S1 in the supplemental material). After removing 54,019 sequences for being identified as chimeras, 66,072 sequences that were identified as being from chloroplasts, and 37 sequences identified as being from mitochondria, the final data set contained 10,038,168 reads. One hundred twenty-three ducks tested negative for IAV infection (i.e., “IAV^−^”), and 177 samples screened positive for IAV (i.e., “IAV^+^”). Of the IAV^+^ samples, 36 unique subtypes were represented, including 12 HAs and 9 NAs (Fig. 2). Four individuals were infected with two HAs, six individuals were infected with two NAs, and one additional individual was infected with two HAs and two NAs.

**TABLE 1:** Duck species and influenza infection summary

Duck species	*n*	Influenza virus status	
		IAV^−^	IAV^+^
All ducks	300	123	177
*A. acuta* (northern pintail)	31	20	11
*A. americana* (American wigeon)	25	10	15
*A. carolinensis* (green-winged teal)	19	10	9
*A. clypeata* (northern shoveler)	57	28	29
*A. platyrhynchos* (mallard)	168	55	113

10.1128/mSphere.00382-18.10 TABLE S1 Relative abundances for bacterial phyla in all samples. Download Table S1, XLSX file, 0.1 MB. Copyright © 2018 Hird et al. 2018 Hird et al. This content is distributed under the terms of the Creative Commons Attribution 4.0 International license.

### Taxonomic diversity.

**FIG 3:**
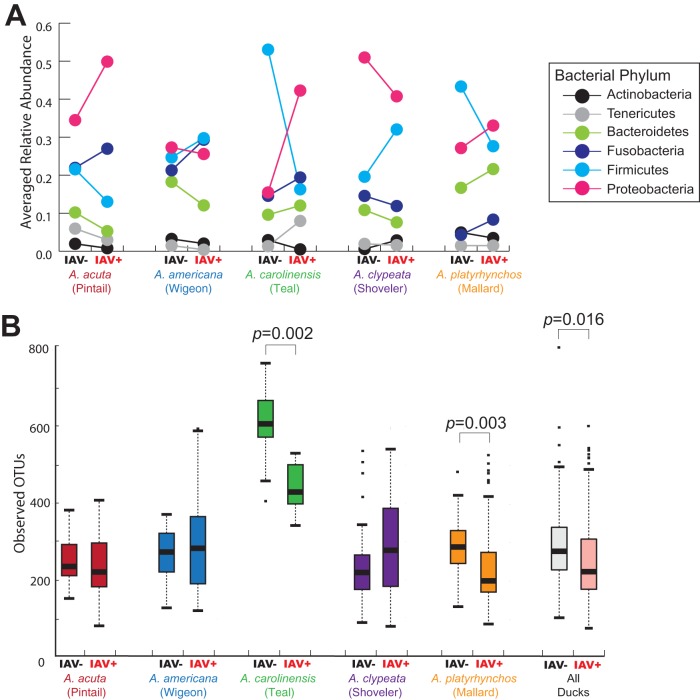
(A) Relative abundance of the six dominant bacterial phyla identified by the RDP classifier in 16S rRNA gene sequences per bird species, broken down by influenza virus status. Note the general lack of pattern between IAV^+^ and IAV^−^ birds across species. The exception is that green-winged teals and mallards show same directionality for all phyla and northern shovelers show the opposite directionality from the green-winged teals and mallards. (B) Bar and whisker plot showing one measure of alpha diversity (observed OTUs) between IAV^−^ and IAV^+^ individuals in each species. Note that there is no consistent pattern across species.

Six bacterial phyla were found in all 124 IAV^−^ birds: *Firmicutes* (average relative abundance, 33.7%), *Proteobacteria* (32.7%), *Bacteroidetes* (13.8%), *Fusobacteria* (11.6%), *Actinobacteria* (3.1%), and *Tenericutes* (2.2%) (Fig. 3A). Sequences unable to be assigned to a bacterial phylum comprised an average of 1.6% of each sample. Another 24 phyla were found in at least two of the IAV^−^ birds (Table S1).

We were interested in whether all birds showed the same relationship to IAV infection. This was easily viewed at the phylum level (Fig. 3A); within each host species, each IAV infection status had dramatically different relative abundances of bacterial phyla. However, there was no consistent pattern to the direction of the differences between IAV^−^ and IAV^+^ birds across species.

### Alpha diversity.

We used three measures of alpha diversity (Chao1, observed OTUs, and phylogenetic diversity [PD]; Table S2) to examine whether IAV^+^ individuals had a consistent difference in alpha diversity. Generally, all three measures were consistent within a species at *P < *0.05 (exception: Anas carolinensis). Only Anas platyrhynchos and A. carolinensis showed a significant decrease in alpha diversity in the IAV^+^ individuals. The other species were not significantly different (and Anas clypeata even showed a nonsignificant trend of increased alpha diversity in IAV^+^ individuals, Fig. 3B).

10.1128/mSphere.00382-18.11 TABLE S2 Alpha diversity data. Download Table S2, XLSX file, 0.03 MB. Copyright © 2018 Hird et al. 2018 Hird et al. This content is distributed under the terms of the Creative Commons Attribution 4.0 International license.

### Beta diversity.

**FIG 4:**
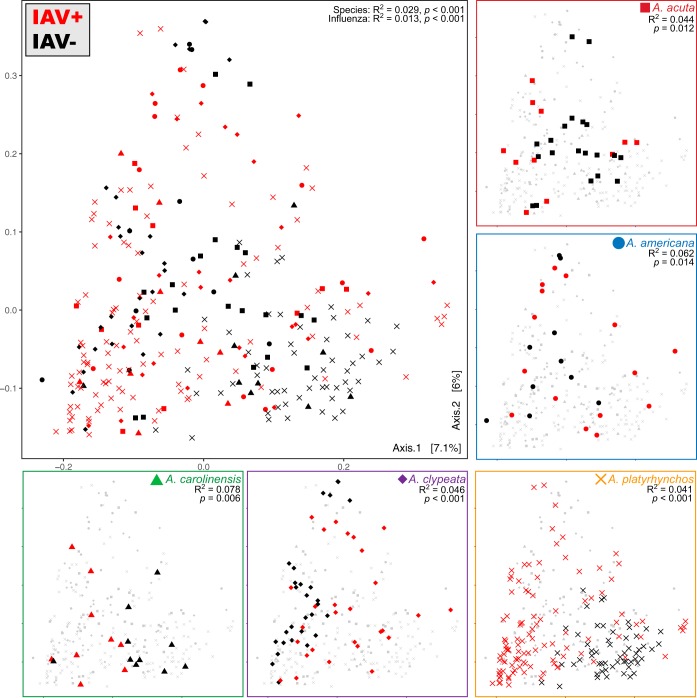
Principal-coordinate analysis of unweighted UniFrac distances (a phylogenetic distance metric) between all samples. Largest panel shows all ducks (species denoted by shape); IAV^+^ (red) versus IAV^−^ (black) samples. Smaller panels highlight each species and are still colored by influenza virus status. Adonis results indicate effect size (*R*^2^) and significance of influenza virus as an explanatory variable for each species; Adonis results for the categories “species” and “influenza status” are shown for all ducks.

To examine how IAV infection correlated to the microbial communities (as a whole), we performed beta diversity analyses using unweighted UniFrac distance matrices (Fig. 4), which were similar in signal to beta diversity calculated with Bray-Curtis dissimilarities and weighted UniFrac distances (Fig. S1). Microbiomes did not strongly group by host species, and although PERMANOVA tests (via Adonis function in vegan package in R) for statistical differences in group centroids were significant for the combined data set (*R*^2^ = 0.029, *P < *0.001), the dispersions of the groups were also significantly different (unweighted UniFrac, *P = *0.003; weighted UniFrac, *P = *0.002), indicating that the significance of the centroid differences may be inflated due to the different dispersions of the groups.

10.1128/mSphere.00382-18.8 FIG S1 NMDS of entire data set using Bray-Curtis and weighted UniFrac distance metrics. Download FIG S1, PDF file, 0.1 MB. Copyright © 2018 Hird et al. 2018 Hird et al. This content is distributed under the terms of the Creative Commons Attribution 4.0 International license.

Influenza virus was always a significant factor (*P < *0.02) with low effect size (*R*^2^ < 0.1). Here, dispersion tests show insignificant differences between the groups, indicating that differences between IAV^−^ and IAV^+^ individuals are not driven by difference in dispersion of the groups. Age of the bird, sampling locality, and sample collection date were all associated with microbiome, as well as the hemagglutinin subtype (HA), neuraminidase subtype (NA), and HA-NA subtype (HANA, Table 2). Once the mallards were removed from the data set, however, only IAV infection status, species, location, and sample collection date remained significant. Removing mallards also caused species to lose significance in the data set that included only IAV^+^ individuals (Table 2).

**TABLE 2:** Effect size and significance of PERMANOVA tests (via the Adonis function in vegan in R) of the metadata in our data set^*a*^

Variable	Full data set						Mallards (*A. platyrhynchos*) removed						Individual species									
	All birds (*n* = 291)		IAV^−^ birds (*n* = 123)		IAV^+^ birds (*n* = 168)		All birds (*n* = 132)		IAV^−^ birds (*n* = 68)		IAV^+^ birds (*n* = 77)		*A. acuta* (*n* = 31)		*A. americana* (*n* = 25)		*A. carolinensis* (*n* = 19)		*A. clypeata* (*n* = 56)		*A. platyrhynchos* (*n* = 160)	
	*R*^2^	*P*	*R*^2^	*P*	*R*^2^	*P*	*R*^2^	*P*	*R*^2^	*P*	*R*^2^	*P*	*R*^2^	*P*	*R*^2^	*P*	*R*^2^	*P*	*R*^2^	*P*	*R*^2^	*P*
Influenza	0.0129	**0.001**	N/A		N/A		0.01667	**0.001**	N/A		N/A		0.04343	0.017	0.06465	0.016	0.0758	0.013	0.04896	**0.001**	0.04188	**0.001**
Species	0.02922	**0.001**	0.10223	**0.001**	0.03789	**0.001**	0.03622	**0.001**	0.08533	**0.001**	0.08979	0.048	N/A		N/A		N/A		N/A		N/A	
Location	0.05167	**0.001**	0.10045	**0.001**	0.07831	**0.001**	0.03274	**0.001**	0.04371	**0.001**	0.04283	**0.001**	0.07724	0.042	0.04338	0.32	0.0882	*0.002*	0.01925	0.269	0.06649	**0.001**
S_SY	0.07547	**0.001**	0.12567	**0.001**	0.10072	**0.001**	0.06597	**0.001**	0.0523	**0.001**	0.06867	*0.004*	0.0878	**0.001**	0.19398	0.026	0.19542	0.006	0.1034	**0.001**	0.0707	**0.001**
HA	0.08397	**0.001**	N/A		0.12199	**0.001**	0.11116	0.007	N/A		0.17928	0.192	0.15134	0.025	0.17336	0.231	0.23895	0.101	0.22263	*0.003*	0.12914	**0.001**
NA	0.06951	**0.001**	N/A		0.09799	**0.001**	0.10974	0.009	N/A		0.16779	0.13	0.10435	0.274	0.17594	0.172	0.29091	0.139	0.21843	0.02	0.12644	**0.001**
HANA	0.15919	**0.001**	N/A		0.25513	**0.001**	0.18245	0.117	N/A		0.32316	0.3543	0.17812	0.108	0.2127	0.311	0.3449	0.212	0.33222	0.286	0.21849	**0.001**
Sex	0.01051	*0.002*	0.0253	*0.002*	0.01395	0.078	0.01685	0.161	0.03759	0.014	0.02461	0.736	0.0426	0.025	0.07903	0.642	0.10573	0.728	0.03732	0.355	0.01561	0.033

a Tests were conducted on unweighted UniFrac distance matrices that were subsampled in various ways. First column is the effect size (*R*^2^), and second is the *P* value. *P* values ≤ 0.001 are indicated in bold, and those less than 0.005 are indicated in italic. N/A, not available.

A RandomForest classifier was used to learn microbiome types from known samples and then assign unknown samples to different categories. We assigned unknown individuals within species to infection status with the following out-of-box (OOB) error rates: Anas acuta (northern pintail) = 26.6%, Anas americana (American wigeon) = 28%, A. carolinensis (green-winged teal) = 21.0%, A. clypeata (northern shoveler) = 10.7%, A. platyrhynchos (mallard) = 11.0%.

### Common microbiome.

A common microbiome of nine OTUs was defined as the OTUs that were present in 90% of individuals in each species (Table 3). The OTUs present in 90% of the IAV^+^ birds and the OTUs present in 90% of the IAV^−^ birds were both subsets of the OTUs found in 90% of all species.

**TABLE 3:** The OTUs that were common to 90% of individuals within each species; taxonomic assignments were made using RDP classifier (see text for more details)

Phylum	Class	Order	Family	Genus or species	OTU_id	IAV^*a*^	
						Positive	Negative
*Proteobacteria*	*Gammaproteobacteria*	*Pasteurellales*	*Pasteurellaceae*		New.ReferenceOTU552	+	−
*Proteobacteria*	*Epsilonproteobacteria*	*Campylobacterales*	*Campylobacteraceae*	*Campylobacter*	284123	+	
*Proteobacteria*	*Epsilonproteobacteria*	*Campylobacterales*	*Campylobacteraceae*	*Campylobacter*	New.ReferenceOTU451	+	−
*Fusobacteria*	*Fusobacteriia*	*Fusobacteriales*	*Leptotrichiaceae*		New.ReferenceOTU81		−
*Fusobacteria*	*Fusobacteriia*	*Fusobacteriales*	*Fusobacteriaceae*	*Fusobacterium*	4439398		
*Firmicutes*	*Clostridia*	*Clostridiales*	*Veillonellaceae*	*Veillonella dispar*	New.ReferenceOTU568		−
*Firmicutes*	*Clostridia*	*Clostridiales*			New.ReferenceOTU701	+	−
*Bacteroidetes*	*Flavobacteriia*	*Flavobacteriales*	*Flavobacteriaceae*		1122504	+	−
*Bacteroidetes*	*Bacteroidia*	*Bacteroidales*	*Porphyromonadaceae*	*Parabacteroides*	New.ReferenceOTU712		−

a IAV, whether that OTU was also found in 90% of all IAV^+^ or IAV^−^ birds.

To further examine the phylogenetic placement of these OTUs, we searched representative sequences from the OTUs using *blastn* against the NCBI nr and 16S rRNA gene databases (which include additional sequences not included in the database used for the taxonomic annotation shown in Table 3) and also constructed phylogenetic trees based on the blast matches. Four of these OTUs had 100% identical matches to sequences in the NCBI nr database. New.ReferenceOTU451 was found to be 100% identical to a single clone isolated from coastal surface waters and fell within the Campylobacter canadensis. OTU 4439398 was 100% identical to an uncultured bacterium from a human ileum (found in the nr database) and in phylogenetic analysis of sequence hits from the 16S rRNA gene database was found to be sister to two sequences belonging to Fusobacterium mortiferum (48). New.ReferenceOTU712 was 100% identical to a sequence found in a study of Canada Goose feces (49). OTU1122504 was 100% identical to Flavobacterium ceti, as well as many uncultured *Flavobacterium* clones. The remaining OTUs did not have any 100% matches to any sequences in either database, but phylogenetic analysis of the matches provides some additional insight into their placement. New.ReferenceOTU552 was sister to a clade containing *Pasteurellaceae* bacteria and uncultured bacteria (see Fig. S2A in the supplemental material). OTU 284123 fell within a clade of *Campylobacter* and was sister to Campylobacter cuniculorum (Fig. S2B). New.ReferenceOTU81 fell within a clade containing Streptobacillus hongkongensis, Leptotrichia goodfellowii, and Sebaldella termitidis and was sister to a clade containing Sneathia sanguinegens, several *Streptobacillus* species, and Oceanivirga salmonicida (Fig. S2C). New.ReferenceOTU568 was sister to a clade containing several *Veillonella* species as well as Megasphaera micronuciformis (Fig. S2D). This clade belonged to a larger clade containing more *Veillonella* and *Dialister* species. New.ReferenceOTU701 was sister to a large clade within *Firmicutes* containing many species (Fig. S2E).

10.1128/mSphere.00382-18.9 FIG S2 NCBI Blast to Tree information for the poorly identified common OTUs. Download FIG S2, PDF file, 0.8 MB. Copyright © 2018 Hird et al. 2018 Hird et al. This content is distributed under the terms of the Creative Commons Attribution 4.0 International license.

### Differentially abundant OTUs.

**FIG 5:**
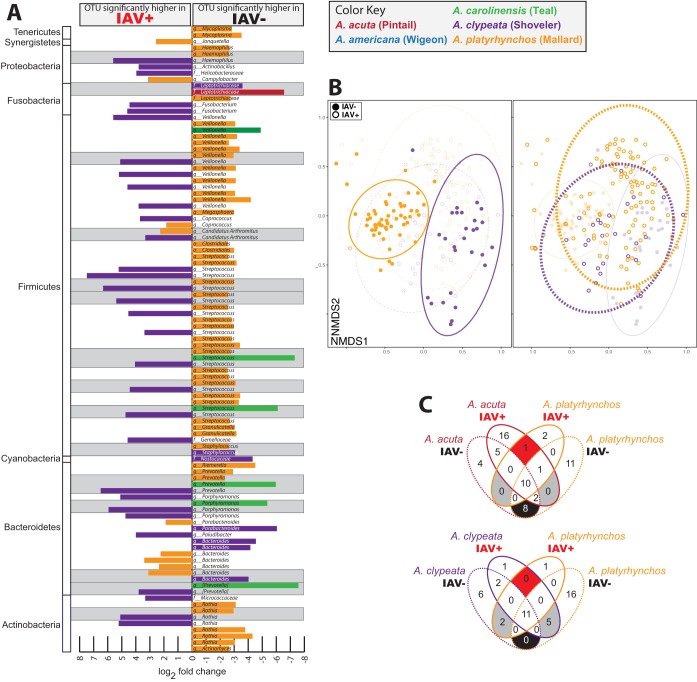
Northern shovelers may have unique microbiome differences with respect to IAV infection, relative to the other duck species. (A) Differentially abundant OTUs within each species. Bars indicate individual OTUs belonging to the taxa indicated by the bar at top of the figure, where each column going up indicates an OTU significantly higher in IAV^+^ birds within a species and columns going down indicate OTUs significantly higher in IAV^−^ birds. Color indicates bird species. Gray bars highlight where an identical OTU was found to be significant in more than one species, with the highest taxonomic resolution of these OTUs numbered and labeled across the bottom. (B) NMDS ordination of Bray-Curtis distances on northern shoveler and mallard samples only. Left panel highlights the IAV^−^ ducks and includes the multivariate *t*-distributed ellipses; right panel highlights the IAV^+^ ducks. (C) Venn diagrams of the OTUs found in 90% of the IAV^+^ and IAV^−^ individuals for mallards and two other species. (Upper) Mallard OTUs compared to northern pintail OTUs, showing that there are some OTUs that overlap in the IAV^+^/IAV^+^ region and in the IAV^−^/IAV^−^ region with no OTUs in the IAV^+^/IAV^−^ regions. These data meet the expectation that there is a similar infection-based pattern to the OTUs in these two species. (Lower) Mallard OTUs compared to northern shoveler OTUs, showing that none of the previous expectations are met and the IAV^+^/IAV^−^ regions contain several OTUs.

Within species, we detected many OTUs that were differentially abundant between IAV^+^ and IAV^−^ birds (Fig. 5A). Compared across the five species, IAV^+^ northern shovelers and IAV^−^ mallards contained many of the same significant taxa/OTUs. Many OTUs were detected that were significantly associated with infection status in more than one species. One pattern worth noting is that northern shovelers and mallards appear to share many OTUs that are significantly associated with flu status but in opposite directions: e.g., box 1 on Fig. 5 depicts a Rothia mucilaginosa OTU that is significantly more abundant in IAV^+^ northern shovelers and IAV^−^ mallards.

### Mallard and northern shoveler comparisons.

We focused next on just the mallards and northern shovelers, as they had the highest sample sizes and to minimize noise in the data. A RandomForest classifier was used to learn microbiome types from known samples and then assign unknown samples to different categories. The classifier had an out-of-box (OOB) error rate of 2.61% for assigning IAV^−^ birds to species and an OOB error rate of 16.5% for IAV^+^ birds. An ordination of just the mallards and northern shovelers shows that IAV^−^ individuals comprise distinct clusters, whereas the IAV^+^ birds are largely overlapping (Fig. 5B). To further look at the differences between northern shovelers and the other species, we created Venn diagrams of the OTUs present in 90% of the IAV^+^ and IAV^−^ individuals within each species (Fig. 5C).

## DISCUSSION

The interactions among a host organism and its microbiome and viral pathogens are complex and likely of evolutionary relevance. Here, we have described the IAV^−^ and IAV^+^ cloacal microbiomes of five *Anas* ducks and found major differences between individuals of different infection statuses within a species, yet little commonality to those differences across species. The communities were largely comprised of six dominant bacterial phyla: *Proteobacteria* and *Firmicutes* together constituted greater than 50% of the bacteria in all bird species and infection statuses. These two phyla have previously been associated with avian microbiomes, including avian intestinal samples (e.g., references 45 and 50) and cloacal swabs specifically (47, 51). *Fusobacteria*, *Actinobacteria*, *Tenericutes*, and *Bacteroidetes* were the other dominant phyla.

The IAV^−^ and IAV^+^ birds within each species showed dramatically different microbial community composition (Fig. 3A). Within each species, infection status explained between 4 and 7% of the microbiome community variation (*P* values < 0.017). Analysis of all birds together showed that infection status explained 1.29% of the variation (*P = *0.001); however, when mallards were removed due to suspicion that their large sample size might be affecting the significance of the tests, infection status continued to have a small (*R*^2^ = 1.66%) but significant (*P = *0.001) association. There were no within-species differences between IAV^−^ and IAV^+^ individuals that are shared across all five species. For example, alpha diversity is significantly lower in the IAV^+^ individuals for mallards and green-winged teals, whereas no significant difference was found for the other three species. In fact, there was a nonsignificant increase in alpha diversity in the IAV^+^ northern shovelers.

Northern shovelers, who are the only filter feeders in the data set, appeared to show an opposite signal relative to the other ducks in a variety of metrics. For example, *Fusobacteria* were higher in the IAV^+^ individuals within each species except the northern shovelers and many of the OTUs that were significantly higher in one infection status in northern shovelers showed the opposite infection status in the other species. When we compared just the mallards and the northern shovelers, the IAV^−^ individuals from both species were distinct, whereas the IAV^+^ individuals were largely overlapping. This was corroborated by the random forest analysis that revealed a higher rate of correct assignment to species in the IAV^−^ birds (2.6% error) than the IAV^+^ birds (16.5% error). In the data sets including all five species, host species was significant (*P = *0.001) in both the IAV^−^ (*R*^2^ = 10.2%) and IAV^+^ data sets (*R*^2^ = 3.7%); however, removal of the mallards caused the effect size in IAV^−^ birds to decrease to 8.5% (*P = *0.001) and in the IAV^+^ data set to increase (*R*^2^ = 8.9%) and become nonsignificant (*P = *0.048). We take this to mean that mallards may have been driving the species signal in the IAV^+^ data set and that there is a much stronger species-level association in the microbiome of IAV^−^ individuals.

**FIG 6:**
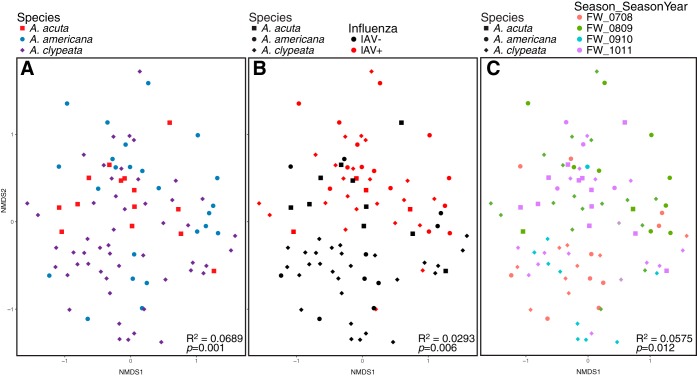
A single NMDS plot of Bray-Curtis dissimilarity of 16S rRNA gene sequences from cloacal swab of ducks from a single locality. Panels highlight ducks of different species (A), IAV infection status (B), and time collected (C).

In addition to the infection status and host species being significantly associated with the metadata, sampling locality (*R*^2^ = 5.1%) and time (season and year, *R*^2^ = 7.5%) were all significantly associated with the microbiomes (*P = *0.001) and remained significant even after removal of the mallards (although effect sizes decreased by ∼1%, Table 2). This is unsurprising, given the strong *a priori* correlations between IAV subtype, season and year sampled, and host species collected at the various localities, as well as the known interactions between these variables, e.g., references 6, 11, 27, and 38), including specifically in mallards (52). Sampling locality can be more important thant ecology or phylogeny for shaping the avian microbiome (45), and we may be seeing this in the ducks as well. When we analyzed three species with both infection statuses from a single locality (Fig. 6), species had both the highest effect size (6.89%) and the lowest *P* value (0.001). Season/year that the samples were collected had the next highest effect size (5.75%, *P = *0.12); IAV infection status had the lowest effect size (2.93%) and a low *P* value (*P = *0.006).

The significance of IAV subtype on the microbiome remains unknown; our data set contained 36 IAV subtypes, with generally low sample sizes for any single HA or NA subtype. Additionally, the subtypes were correlated to sampling site, time of collection, and species. However, HA subtype, NA subtype, and HA-NA subtype were all significantly associated with the microbiome in the full data set, with subtype (HANA) showing an effect size of 25% in the IAV^+^ birds (*P = *0.001). The significance of all three rose to greater than 0.13, though, when the mallards were removed. Subtypes were not significantly correlated with the microbiome within any of the species except the mallard (HA, NA, and HANA, *P = *0.001) and the association between the northern shoveler and the HA subtype (*P = *0.003). This indicates that HA, NA, and HA-NA subtype may be correlated with the microbiome; however, how significant that association is cannot be established.

Sample size may be essential for determining which metadata are associated with the microbiome across infection statuses—species with larger sample sizes (mallards and northern shovelers) had more categorical data significantly associated with them than the lower-sample-size species (Table 2). This is unsurprising, as the complex interactions between these likely confounded variables make for a challenging statistical problem. We hypothesize that increasing the sample size for green-winged teals, northern pintails, and American wigeons would likely uncover more statistically robust associations between birds and their microbiomes.

Do these data bear on the causative hypotheses? All the data are congruent with the “IAV→ΔMB” hypothesis, particularly the fact that the strength of the host species association is higher in IAV^−^ birds than in IAV^+^ birds. This implies that there is a species-level microbiome for these duck species that deteriorates during IAV infection. It also implies that over time, ducks may return to their “species state,” which is predicted by the “IAV→ΔMB” hypothesis. Our results, however, are also compatible with the “ΔMB→IAV” hypothesis: including all dysbiotic states as one would cause the dispersion of the IAV^+^ groups to be large and the IAV^−^ groups to be smaller. It may also be true that whom a duck associates with (or is simply near) may influence its likelihood of IAV infection, including the subtype. Mixed species groups may have increased exposure to flu but also have a more similar microbiome. Time series data in these wild reservoir species would be ideal to determine a causative role of IAV on dysbiosis or dysbiosis on IAV susceptibility.

## MATERIALS AND METHODS

### Sampling.

Samples were collected from both living ducks (all mallard samples) and those that were recently deceased (<2 h) (for all other species). Samples were collected via swabs of cloaca. Total number of cloacal swabs for each species were as follows: 31 northern pintails (Anas acuta), 25 American wigeons (Anas americana), 19 green-winged teals (Anas carolinensis), 57 northern shovelers (Anas clypeata), and 168 mallards (Anas platyrhynchos), including the 122 juvenile individuals from the work of Ganz et al. (47). Detailed information about the samples is found in Table 1 and Table S3. Swabs were placed in separate vials containing 2 ml of ice-cold virus transport medium (VTM; medium 199 with Earle’s salts, l-glutamine, and sodium bicarbonate, plus 2 mU/liter penicillin G, 200 mg/liter streptomycin, 2 mU/liter polymyxin B, 250 mg/liter gentamicin, 0.5 mU/liter nystatin, 60 mg/liter ofloxacin, 200 mg/liter sulfamethoxazole, and 0.5% bovine serum albumin V). The samples were transported on ice to the laboratory where they were stored at −80°C. Samples underwent one or more freeze-thaw cycles as aliquots were removed for influenza virus testing prior to microbiome analysis.

10.1128/mSphere.00382-18.12 TABLE S3 Sample information. Download Table S3, XLSX file, 0.1 MB. Copyright © 2018 Hird et al. 2018 Hird et al. This content is distributed under the terms of the Creative Commons Attribution 4.0 International license.

### Ethics statement.

All animal research was conducted with the approval of the University of California Davis Animal Care and Use Committee, approval number 19760. The protocols adhere to federal law, specifically the Animal Welfare Act and the Health Research Extension Act of 1985.

### Influenza virus screening and typing.

Samples were screened for the influenza virus matrix gene by RT-PCR, viruses were isolated from matrix-positive samples by egg inoculation, and full genome sequences were generated as described in reference 53. Samples were classified as “negative” if they (i) did not yield virus on egg inoculation and (ii) were matrix RT-PCR negative.

### DNA extraction and 16S rRNA gene sequencing.

DNA extraction and sequencing of the V4 region of 16S rRNA genes were performed in the same manner as in reference 47. DNA was extracted from cloacal swabs using the Mo Bio PowerSoil 96-well soil DNA isolation kit (Carlsbad, CA). Samples in 96-well plates were incubated at 65°C for ten minutes after addition of C1 solution. Each 96-well plate was then vortexed for 3 min using a plate shaker on high (2,600 rpm), and then the standard kit protocol was followed. After elution, DNA was quantified using Qubit fluorometric quantitation (Invitrogen, South San Francisco, CA). We used nested PCR to amplify and sequence the 16S rRNA gene. DNA was characterized for bacterial diversity based on the V4 region of the16S rRNA gene following the methods of reference 54. When possible, 1.0 to 5.0 μl of template DNA was used for PCR. However, due to low DNA concentrations for some samples, bacterial DNA was amplified by a two-step PCR enrichment using the primers 27F−YM+3 and 1492R to target V1 to V4 of the 16S rRNA gene (55). The 7-fold-degenerate primer 27f−YM+3 is composed of four parts 27f−YM (AGAGTTTGATYMTGGCTCAG), plus one part each of primers specific for the amplification of *Bifidobacteriaceae*, *Borrelia*, and *Chlamydiales* sequences (55). For the second PCR, primers used were the bacteria/archaeal primers 515F/806R (54) modified by addition of Illumina adaptor and an in-house barcode system (described in reference 56). After amplification. magnetic beads (Agencourt AMPure XP, Beckman Coulter, Indianapolis, IN) were used to clean PCR mixtures. Amplicons were quantified and characterized using Qubit fluorometric quantitation, qPCR, and the Bioanalyzer (Agilent Technologies, Santa Clara, CA) prior to sequencing. Libraries were sequenced using an Illumina MiSeq system, generating 250-bp paired-end amplicon reads. The amplicon data were multiplexed using dual barcode combinations for each sample.

### Data analysis.

We used a custom script (available in a GitHub repository, https://github.com/gjospin/scripts/blob/master/Demul_trim_prep.pl) to assign each pair of reads to their respective samples when parsing the raw data. This script allows for 1-bp difference per barcode. The paired reads were then aligned, and a consensus was computed using FLASH (57) with maximum overlap of 120 and a minimum overlap of 70 (other parameters were left as default). The custom script automatically demultiplexes the data into fastq files, executes FLASH, and parses its results to reformat the sequences with appropriate naming conventions for Quantitative Insights in Microbial Ecology (QIIME v.1.9.1 [58]) in fasta format; QIIME and the R package phyloseq (59) were subsequently used for most analyses.

First, representative operational taxonomic units (OTUs) were chosen by *de novo* clustering of sequences at the >97% identity level, the taxonomic composition of each cluster was assigned with RDP classifier, and an OTU table was constructed for each sample using QIIME’s pick_otus_through_otu_table.py script. Alpha diversity (observed OTUs, phylogenetic diversity, Chao1) was estimated using the core_diversity_analyses.py script. Pairwise distance matrices were constructed for unweighted UniFrac distance (60), weighted UniFrac distance, and Bray-Curtis dissimilarity for beta diversity analyses.

Differentially abundant OTUs were inferred using the program DESeq2 (61) through the program phyloseq. Each species was analyzed separately for OTUs that were significantly higher (adjusted *P* value < 0.001) in either IAV^+^ or IAV^−^ individuals. Only OTUs with a log fold increase greater than 2 were retained. The R package “randomForest” (62) was used to perform machine learning classifications within species and infection statuses.

### Accession number(s).

Sequence data have been uploaded to the NCBI SRA under BioProject ID PRJNA464410.
