# Supplementary material for: The Cloacal Microbiome of Five Wild Duck Species Varies by Species and Influenza A Virus Infection Status
Source: mSphere. 2018 Oct 24;3(5):e00382-18. doi: 10.1128/mSphere.00382-18 (PMC6200988; doi:10.1128/mSphere.00382-18)

Figure S1: Beta diversity using additional distance metrics of the full dataset.

A: NMDS of Bray-Curtis dissimilarity distances.

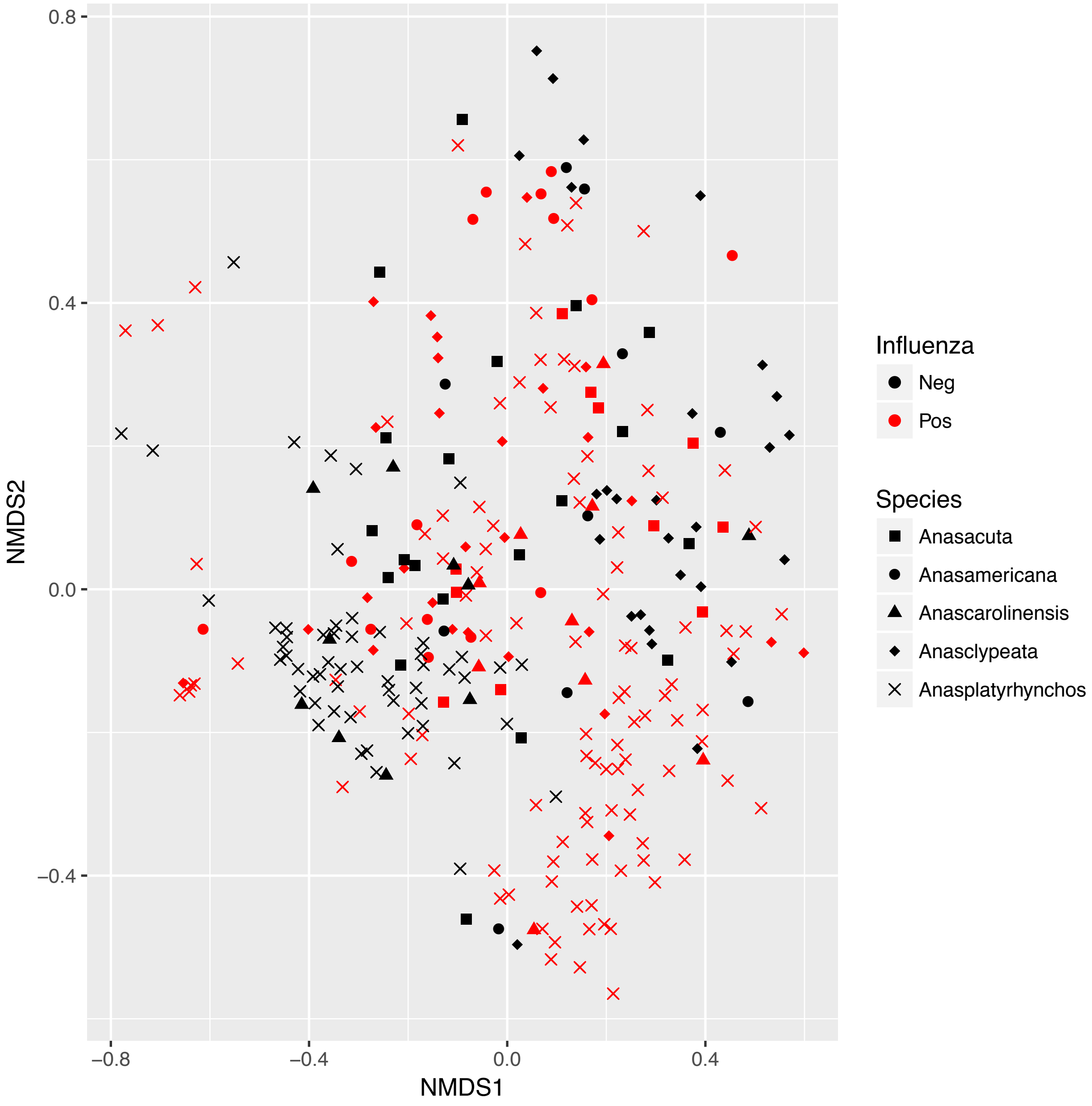

B: NMDS of weighted UniFrac distances.

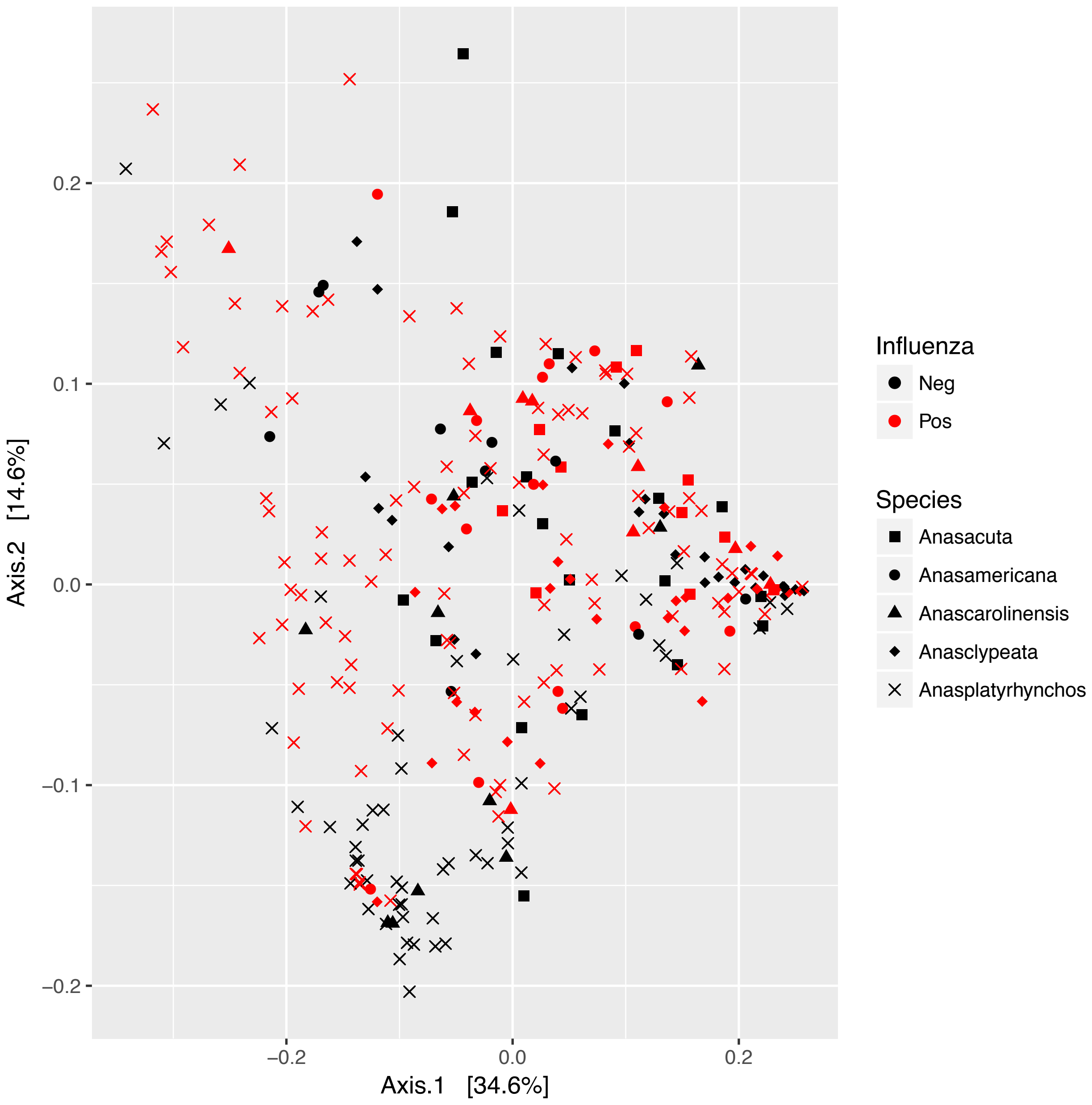

Supplement: FIG S1 [file sph005182671sf1.pdf]
