# Supplementary material for: The Cloacal Microbiome of Five Wild Duck Species Varies by Species and Influenza A Virus Infection Status
Source: mSphere. 2018 Oct 24;3(5):e00382-18. doi: 10.1128/mSphere.00382-18 (PMC6200988; doi:10.1128/mSphere.00382-18)

Supplemental figures for Hird et al. 2018. The cloacal microbiome and influenza A virus infection in five wild reservoir species (ducks, genus *Anas*)

Figure S2. "Blast to tree" figures from NCBI of the weakly identified OTUs.

A. New.ReferenceOTU552

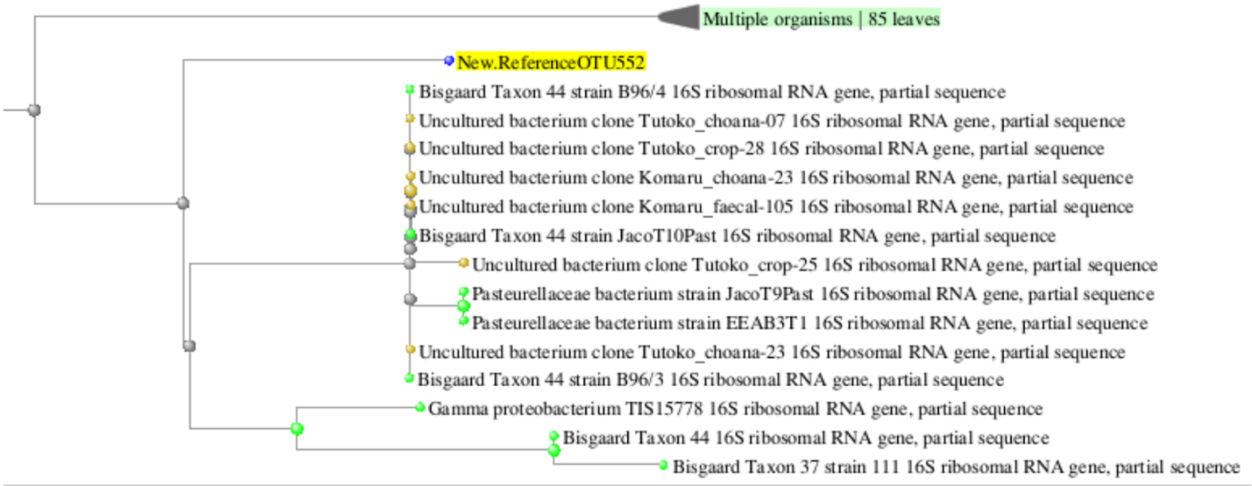

B. OTU284123

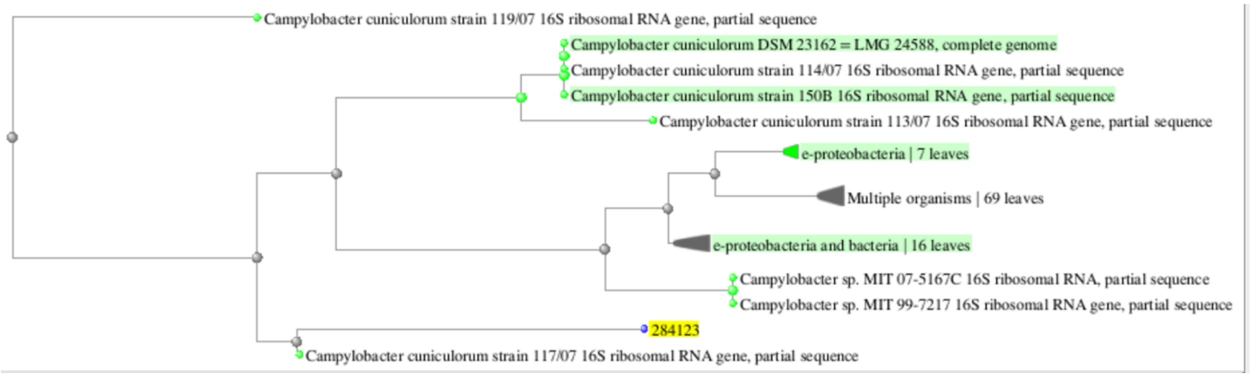

C. New.ReferenceOTU81

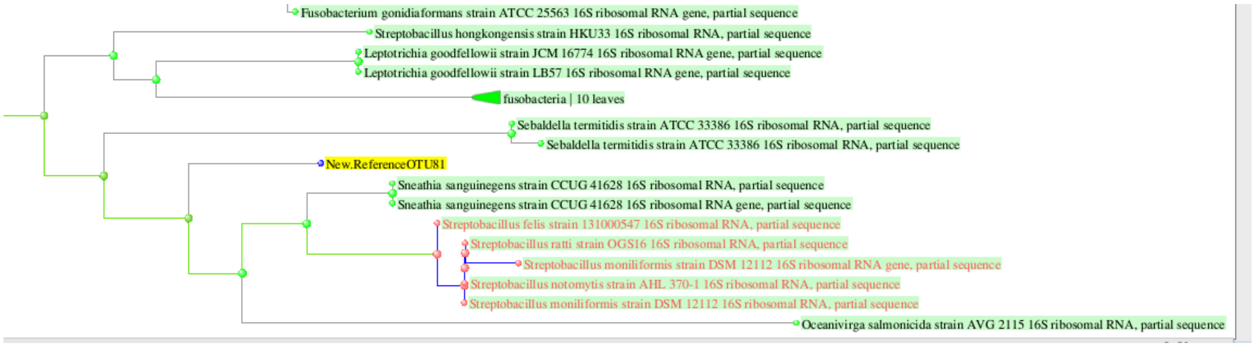

D. New.ReferenceOTU568

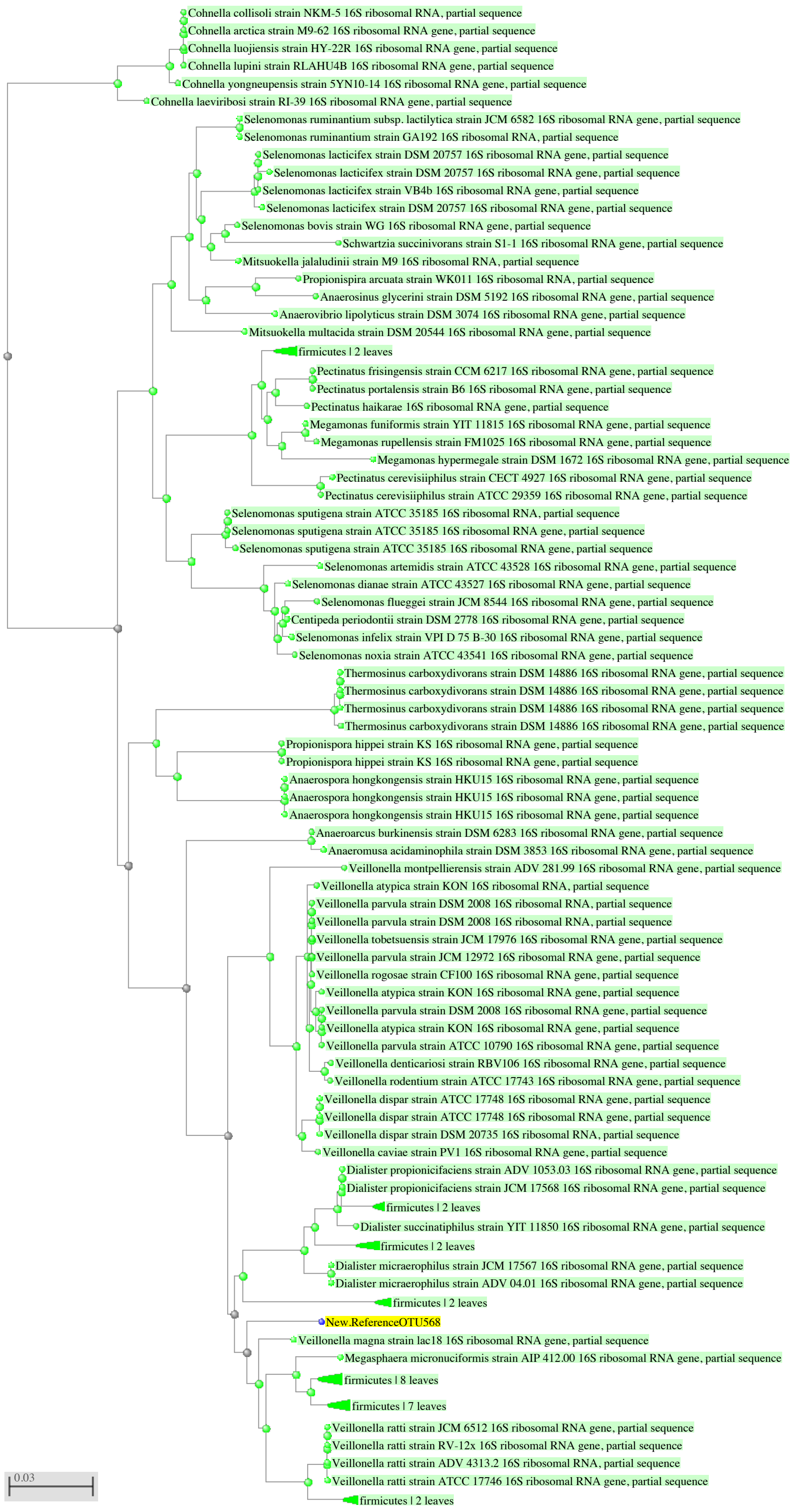
Cohnella collisoli strain NKM-5 16S ribosomal RNA, partial sequence
Cohnella arctica strain M9-62 16S ribosomal RNA gene, partial sequence
Cohnella luojiensis strain HY-22R 16S ribosomal RNA gene, partial sequence
Cohnella lupini strain RLAHU4B 16S ribosomal RNA gene, partial sequence
Cohnella yongneupensis strain 5YN10-14 16S ribosomal RNA gene, partial sequence
Cohnella laeviribosi strain RI-39 16S ribosomal RNA gene, partial sequence
Selenomonas ruminantium subsp. lactilytica strain JCM 6582 16S ribosomal RNA gene, partial sequence
Selenomonas ruminantium strain GA192 16S ribosomal RNA, partial sequence
Selenomonas lacticifex strain DSM 20757 16S ribosomal RNA gene, partial sequence
Selenomonas lacticifex strain DSM 20757 16S ribosomal RNA gene, partial sequence
Selenomonas lacticifex strain VB4b 16S ribosomal RNA gene, partial sequence
Selenomonas lacticifex strain DSM 20757 16S ribosomal RNA gene, partial sequence
Selenomonas bovis strain WG 16S ribosomal RNA gene, partial sequence
Schwartzia succinivorans strain S1-1 16S ribosomal RNA gene, partial sequence
Mitsuokella jalaludinii strain M9 16S ribosomal RNA, partial sequence
Propionispira arcuata strain WK011 16S ribosomal RNA, partial sequence
Anaerosinus glycerini strain DSM 5192 16S ribosomal RNA gene, partial sequence
Anaerovibrio lipolyticus strain DSM 3074 16S ribosomal RNA gene, partial sequence
Mitsuokella multacida strain DSM 20544 16S ribosomal RNA gene, partial sequence
firmicutes | 2 leaves
Pectinatus frisingensis strain CCM 6217 16S ribosomal RNA gene, partial sequence
Pectinatus portalensis strain B6 16S ribosomal RNA gene, partial sequence
Pectinatus haikarae 16S ribosomal RNA gene, partial sequence
Megamonas funiformis strain YIT 11815 16S ribosomal RNA gene, partial sequence
Megamonas rupellensis strain FM1025 16S ribosomal RNA gene, partial sequence
Megamonas hypermegale strain DSM 1672 16S ribosomal RNA gene, partial sequence
Pectinatus cerevisiiphilus strain CECT 4927 16S ribosomal RNA gene, partial sequence
Pectinatus cerevisiiphilus strain ATCC 29359 16S ribosomal RNA gene, partial sequence
Selenomonas sputigena strain ATCC 35185 16S ribosomal RNA, partial sequence
Selenomonas sputigena strain ATCC 35185 16S ribosomal RNA gene, partial sequence
Selenomonas sputigena strain ATCC 35185 16S ribosomal RNA gene, partial sequence
Selenomonas artemidis strain ATCC 43528 16S ribosomal RNA gene, partial sequence
Selenomonas dianae strain ATCC 43527 16S ribosomal RNA gene, partial sequence
Selenomonas flueggei strain JCM 8544 16S ribosomal RNA gene, partial sequence
Centipeda periodontii strain DSM 2778 16S ribosomal RNA gene, partial sequence
Selenomonas infelix strain VPI D 75 B-30 16S ribosomal RNA gene, partial sequence
Selenomonas noxia strain ATCC 43541 16S ribosomal RNA gene, partial sequence
Thermosinus carboxydivorans strain DSM 14886 16S ribosomal RNA gene, partial sequence
Thermosinus carboxydivorans strain DSM 14886 16S ribosomal RNA gene, partial sequence
Thermosinus carboxydivorans strain DSM 14886 16S ribosomal RNA gene, partial sequence
Thermosinus carboxydivorans strain DSM 14886 16S ribosomal RNA gene, partial sequence
Propionispora hippei strain KS 16S ribosomal RNA gene, partial sequence
Propionispora hippei strain KS 16S ribosomal RNA gene, partial sequence
Anaerospora hongkongensis strain HKU15 16S ribosomal RNA gene, partial sequence
Anaerospora hongkongensis strain HKU15 16S ribosomal RNA gene, partial sequence
Anaerospora hongkongensis strain HKU15 16S ribosomal RNA gene, partial sequence
Anaeroarcus burkinensis strain DSM 6283 16S ribosomal RNA gene, partial sequence
Anaeromusa acidaminophila strain DSM 3853 16S ribosomal RNA gene, partial sequence
Veillonella montpellierensis strain ADV 281.99 16S ribosomal RNA gene, partial sequence
Veillonella atypica strain KON 16S ribosomal RNA, partial sequence
Veillonella parvula strain DSM 2008 16S ribosomal RNA gene, partial sequence
Veillonella parvula strain DSM 2008 16S ribosomal RNA, partial sequence
Veillonella tobetsuensis strain JCM 17976 16S ribosomal RNA gene, partial sequence
Veillonella parvula strain JCM 12972 16S ribosomal RNA gene, partial sequence
Veillonella rogosae strain CF100 16S ribosomal RNA gene, partial sequence
Veillonella atypica strain KON 16S ribosomal RNA gene, partial sequence
Veillonella parvula strain DSM 2008 16S ribosomal RNA gene, partial sequence
Veillonella atypica strain KON 16S ribosomal RNA gene, partial sequence
Veillonella parvula strain ATCC 10790 16S ribosomal RNA gene, partial sequence
Veillonella denticariosi strain RBV106 16S ribosomal RNA gene, partial sequence
Veillonella rodentium strain ATCC 17743 16S ribosomal RNA gene, partial sequence
Veillonella dispar strain ATCC 17748 16S ribosomal RNA gene, partial sequence
Veillonella dispar strain ATCC 17748 16S ribosomal RNA gene, partial sequence
Veillonella dispar strain DSM 20735 16S ribosomal RNA, partial sequence
Veillonella caviae strain PV1 16S ribosomal RNA gene, partial sequence
Dialister propionicifaciens strain ADV 1053.03 16S ribosomal RNA gene, partial sequence
Dialister propionicifaciens strain JCM 17568 16S ribosomal RNA gene, partial sequence
firmicutes | 2 leaves
Dialister succinatiphilus strain YIT 11850 16S ribosomal RNA gene, partial sequence
firmicutes | 2 leaves
Dialister micraerophilus strain JCM 17567 16S ribosomal RNA gene, partial sequence
Dialister micraerophilus strain ADV 04.01 16S ribosomal RNA gene, partial sequence
firmicutes | 2 leaves
New.ReferenceOTU568
Veillonella magna strain lac18 16S ribosomal RNA gene, partial sequence
Megasphaera micronuciformis strain AIP 412.00 16S ribosomal RNA gene, partial sequence
firmicutes | 8 leaves
firmicutes | 7 leaves
Veillonella ratti strain JCM 6512 16S ribosomal RNA gene, partial sequence
Veillonella ratti strain RV-12x 16S ribosomal RNA gene, partial sequence
Veillonella ratti strain ADV 4313.2 16S ribosomal RNA, partial sequence
Veillonella ratti strain ATCC 17746 16S ribosomal RNA gene, partial sequence
firmicutes | 2 leaves
0.03

E. New.ReferenceOTU701

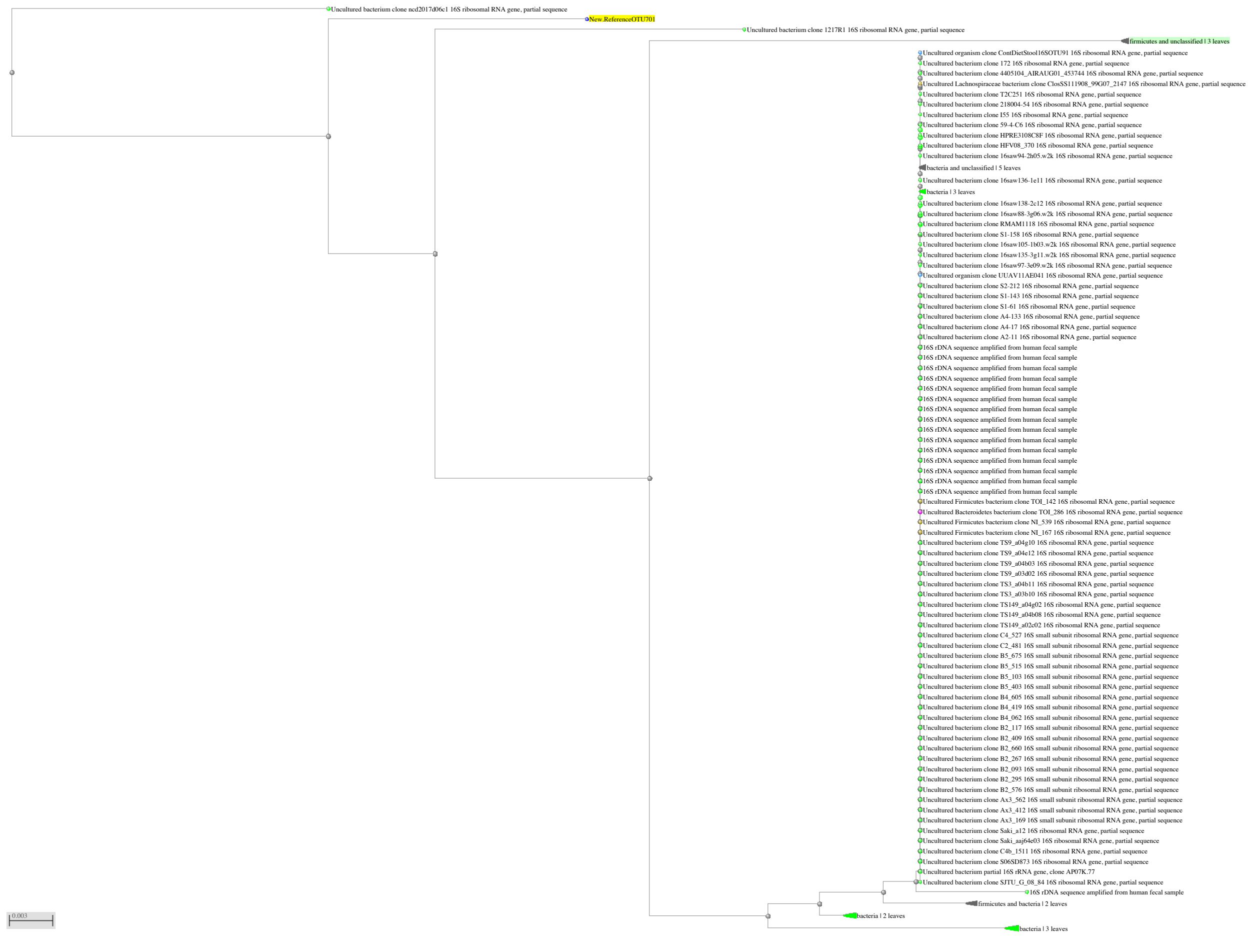

Supplement: FIG S2 [file sph005182671sf2.pdf]
